# Arterial spin labelling and diffusion-weighted imaging in paediatric brain tumours

**DOI:** 10.1016/j.nicl.2019.101696

**Published:** 2019-01-29

**Authors:** Patrick W. Hales, Felice d'Arco, Jessica Cooper, Josef Pfeuffer, Darren Hargrave, Kshitij Mankad, Chris Clark

**Affiliations:** a Developmental Imaging & Biophysics Section, UCL Great Ormond Street Institute of Child Health, London WC1N 1EH, United Kingdom; b Great Ormond Street Children's Hospital, Great Ormond St, London WC1N 3JH, United Kingdom; c Siemens Healthcare GmbH, MR Application Development, Erlangen, Germany

**Keywords:** Arterial spin labeling, Diffusion-weighting imaging, Brain tumour, Paediatrics

## Abstract

**Background:**

Diffusion- and perfusion-weighted MRI are valuable tools for measuring the cellular and vascular properties of brain tumours. This has been well studied in adult patients, however, the biological features of childhood brain tumours are unique, and paediatric-focused studies are less common. We aimed to assess the diagnostic utility of apparent diffusion coefficient (ADC) values derived from diffusion-weighted imaging (DWI) and cerebral blood flow (CBF) values derived from arterial spin labelling (ASL) in paediatric brain tumours.

**Methods:**

We performed a meta-analysis of published studies reporting ADC and ASL-derived CBF values in paediatric brain tumours. Data were combined using a random effects model in order to define typical parameter ranges for different histological tumour subtypes and WHO grades. New data were also acquired in a ‘validation cohort’ at our institution, in which ADC and CBF values in treatment naïve paediatric brain tumour patients were measured, in order to test the validity of the findings from the literature in an un-seen cohort. ADC and CBF quantification was performed by two radiologists via manual placement of tumour regions of interest (ROIs), in addition to an automated approach to tumour ROI placement.

**Results:**

A total of 14 studies met the inclusion criteria for the meta-analysis, constituting data acquired in 542 paediatric patients. Parameters of interest were based on measurements from ROIs placed within the tumour, including mean and minimum ADC values (ADC_ROI-mean_, ADC_ROI-min_) and the maximum CBF value normalised to grey matter (nCBF_ROI-max_). After combination of the literature data, a number of histological tumour subtype groups showed significant differences in ADC values, which were confirmed, where possible, in our validation cohort of 32 patients. In both the meta-analysis and our cohort, diffuse midline glioma was found to be an outlier among high-grade tumour subtypes, with ADC and CBF values more similar to the low-grade tumours. After grouping patients by WHO grade, significant differences in grade groups were found in ADC_ROI-mean_, ADC_ROI-min_, and nCBF_ROI-max_, in both the meta-analysis and our validation cohort. After excluding diffuse midline glioma, optimum thresholds (derived from ROC analysis) for separating low/high-grade tumours were 0.95 × 10^−3^ mm^2^/s (ADC_ROI-mean_), 0.82 × 10^−3^ mm^2^/s (ADC_ROI-min_) and 1.45 (nCBF_ROI-max_). These thresholds were able to identify low/high-grade tumours with 96%, 83%, and 83% accuracy respectively in our validation cohort, and agreed well with the results from the meta-analysis. Diagnostic power was improved by combining ADC and CBF measurements from the same tumour, after which 100% of tumours in our cohort were correctly classified as either low- or high-grade (excluding diffuse midline glioma).

**Conclusion:**

ADC and CBF values are useful for differentiating certain histological subtypes, and separating low- and high-grade paediatric brain tumours. The threshold values presented here are in agreement with previously published studies, as well as a new patient cohort. If ADC and CBF values acquired in the same tumour are combined, the diagnostic accuracy is optimised.

## Highlights

• ADC and CBF values differ as a function of WHO grade in paediatric brain tumours.
• Mean ADC < 0.95 × 10^−3^ mm^2^/s is indicative of a high-grade tumour.
• Max CBF (normalised to grey matter) > 1.45 also indicates a high-grade tumour.
• Combining ADC and CBF values from the same tumour improves the diagnostic power.

## Graphical abstract

**Figure:**
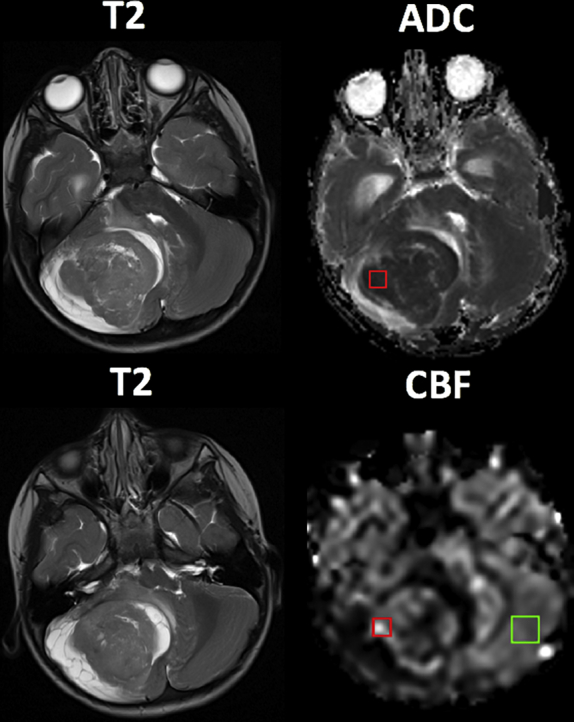

## 1 Introduction

Brain tumours represent the most common solid tumour of childhood, and one of the highest causes of paediatric cancer-related mortality (Pollack, 1994). Magnetic resonance imaging (MRI) plays an essential role in the clinical management of these patients. However, paediatric brain tumours encompass a broad spectrum of histopathological features, and although conventional MRI sequences are well suited to identifying the presence of a tumour, they often fail to provide sufficient sensitivity and specificity regarding its underlying biology (Borja et al., 2013; Peet et al., 2012; Poretti et al., 2012; Poussaint and Rodriguez, 2006).

Advanced MRI techniques, such as diffusion- and perfusion-weighted imaging (DWI and PWI, respectively), provide important additional information regarding the biology and physiology of brain tumours. DWI is the most commonly used of these techniques, and the use of apparent diffusion coefficient (ADC) values to determine cellular density, and to infer histologic subtype and/or malignancy in paediatric brain tumours, has been widely reported (Bull et al., 2012; Chang et al., 2003; Chen et al., 2010; Choudhri et al., 2015; Gimi et al., 2012; Koral et al., 2013; Kralik et al., 2014; Orman et al., 2015.; Poretti et al., 2013; Rumboldt et al., 2006).

Neovascularisation is also considered a key mechanism in tumour growth and malignancy; however, perfusion-based studies in paediatric brain tumours are less frequently reported (Cha, 2006; Dangouloff-Ros et al., 2016; Dangouloff-Ros et al., 2015; Kikuchi et al., 2017; Lobel et al., 2011; Morana et al., 2018; Thompson et al., 2012; Tzika et al., 2004; Yeom et al., 2014). This is due in part to PWI of brain tumours traditionally being performed using dynamic susceptibility contrast (DSC) MRI, which requires injection of a paramagnetic contrast agent via high-flow power injectors and wide-bore intravenous cannulas, which poses technical challenges in young patients (Cha, 2006; Dangouloff-Ros et al., 2016). However, arterial spin labelling (ASL) has emerged as a promising alternative to DSC-MRI in the evaluation of tumour vascularity (Hirai et al., 2011; Warmuth et al., 2003; White et al., 2014). ASL does not require injection of an exogenous contrast agent, making it well suited for paediatric use, and ASL-based studies have shown promising results for the assessment of malignancy in paediatric brain tumours (Dangouloff-Ros et al., 2016; Delgado et al., 2018; Kikuchi et al., 2017; Yeom et al., 2014).

A number of studies have attempted to quantify typical values of ADC or cerebral blood flow (CBF) in common World Health Organisation (WHO) paediatric brain tumour histological subtypes or grades, in order to provide threshold values to aid clinical diagnosis (Bull et al., 2012; Chang et al., 2003; Chen et al., 2010; Choudhri et al., 2015; Dangouloff-Ros et al., 2016; Delgado et al., 2018; Gimi et al., 2012; Kikuchi et al., 2017; Koral et al., 2013; Kralik et al., 2014; Morana et al., 2018; Orman et al., 2015.; Poretti et al., 2013; Rumboldt et al., 2006). Furthermore, following the updated WHO classification of central nervous system tumours in 2016, (Louis et al., 2016) in which the importance of molecular stratification of brain tumours has been recognised, a growing interest in ‘radio-genomics’ has emerged, which aims to define the relationship between imaging features and molecular tumour markers (Kuo and Jamshidi, 2014). Early studies have shown promise in differentiating some brain tumour molecular subtypes non-invasively using this technique, (Figini et al., 2018; Jansen et al., 2018; Liu et al., 2018; Zhou et al., 2018) and, although paediatric-based radio-genomics studies are still in their infancy, promising results have been obtained in identifying molecular subtypes of medulloblastoma using MRI (Dangouloff-Ros et al., 2018; Dasgupta et al., 2019; Perreault et al., 2014).

As paediatric brain tumours represent a rare disease, it is challenging to acquire sufficient data in a single institution to characterise typical ADC and CBF values across the wide spectrum of biology found in these tumours. Furthermore, representative values reported in the literature can vary considerably between studies. A proportion of this variation is due to differences in the method by which representative ADC and CBF values for a given tumour are extracted. Although automated tumour segmentation and analysis techniques may alleviate this issue, (Angulakshmi and Priya, 2017) these are not yet standardised or routinely available in the clinic. As such, most studies rely on manual placement of regions of interest (ROIs) by a radiologist, using a clinical workstation with limited image processing capabilities. The definition of these ROIs tends to vary between studies. Some encompass the entire ‘solid tumour’, (Bull et al., 2012; Calmon et al., 2017; Chen et al., 2010) however more commonly a number of small ROIs (typically 5-75 mm^2^) are hand-placed within the tumour volume, in regions judged to have the lowest ADC or highest CBF values (Chang et al., 2003; Dangouloff-Ros et al., 2016; Dangouloff-Ros et al., 2015; Kikuchi et al., 2017; Koral et al., 2013; Kralik et al., 2014; Morana et al., 2018; Poretti et al., 2013; Rumboldt et al., 2006). This represents a relatively simple and easily implemented method for quantifying tumour ADC and CBF values in the clinic, and it is therefore important to understand the reliability of this technique.

Having acquired a ‘representative sample’ of the tumour by placing an ROI, there is additional variation in which metrics are subsequently reported. The mean ADC value within this ROI (ADC_ROI-mean_) is often used, however, there are a number of studies which suggest the minimum ADC (ADC_ROI-min_) may be superior for differentiating WHO grade, (Jaremko et al., 2010; Kralik et al., 2014; Lee et al., 2008) although this has recently been disputed (Surov et al., 2017). What appears more clear is that, for CBF, the maximum value (rather than the mean) is best for differentiating WHO grade (Aronen et al., 1994; Knopp et al., 1999; Law et al., 2003; Noguchi et al., 2008; Shin et al., 2002; Sugahara et al., 2001; Warmuth et al., 2003; Yeom et al., 2014). However, particularly in paediatrics, it is important that CBF values are normalised to a reference tissue within the patient's brain, in order to account for age and other patient-dependent variations in cerebral perfusion, such as hydrocephalus or anaesthesia (Hales et al., 2014; Yeom et al., 2014). Normal appearing grey matter (GM) is preferred for this, due to the higher signal-to-noise ratio and reduced arterial transit time compared to white matter (van Gelderen et al., 2008; Yeom et al., 2014). This reference ROI is sometimes placed in a fixed anatomical position, such as the contralateral thalamus (Kikuchi et al., 2017) or temporal pole; (Morana et al., 2018) however, in studies which include tumours occurring over a wide range of anatomical locations, a region of normal appearing grey matter contralateral to each individual tumour is often used (Yeom et al., 2014).

The aim of this study was two-fold. Firstly, we performed a meta-analysis of the available literature regarding ADC and CBF values measured in common paediatric brain tumour subtypes, and by combining data from previous studies, aimed to define typical parameter ranges for different histological subtypes and WHO grades. In this study, we focussed on histological rather than molecular subtypes, due to the comparatively large quantity of published data regarding the diffusion and perfusion characteristics of former. Following this, we acquired ADC and CBF maps in a validation cohort of treatment naïve paediatric brain tumour patients at our institution. This cohort was used to (a) assess the reliability of extracting tumour ADC and CBF values by the manual placement of ROIs, (b) determine if conclusions drawn from the meta-analysis were valid in an un-seen data set, and (c) assess the added diagnostic value of combining ADC and CBF measurements from the same tumour, which has not been widely reported in paediatric brain tumours.

## 2 Methods

### 2.1 Meta-analysis

#### 2.1.1 Search strategy

A systematic search was performed in PubMed, using the following search terms:

*(diffusion* OR *ADC* OR *DWI* OR *“arterial spin labelling”* OR *“arterial spin labeling”* OR *ASL)* AND *((paediatric* OR *paediatric* OR *child)* AND *(brain* OR *CNS)* AND *(tumour* OR *tumour* OR *neoplasm))*. In addition, references from selected articles were examined manually for potentially relevant studies which were not identified in the above search.

Our inclusion criteria were as follows: (1) ASL or DWI was used to measure CBF or ADC values respectively, in treatment naïve paediatric brain tumours; (2) subsequent histological confirmation of tumour subtype was performed, with the exception of optic pathway glioma and diffuse midline glioma, in which tissue sampling is not generally used for diagnosis; (3) imaging parameters were obtained from tumour ROIs avoiding large blood vessels, necrotic, cystic and haemorrhagic regions (identified using conventional MRI sequences); (4) studies reported ADC_ROI-mean_, and/or ADC_ROI-min_ values, or maximum normalised CBF values (nCBF_ROI-max_) for the tumour, the latter being normalised to normal appearing grey matter in the same patient.

Studies were excluded based on the following criteria: animal studies, studies which reported patients grouped by WHO grade only; studies reporting normalised ADC values only; ASL studies which did not report CBF_max_ values; studies in which CBF_max_ values were not normalised to normal appearing grey matter; non-paediatric studies.

#### 2.1.2 Data analysis

Reported mean values of ADC_ROI-mean_, ADC_ROI-min_, or nCBF_ROI-max_ for different histological tumour subtypes, along with their standard deviation (SD), were used as individual estimates from each included study. These were combined to produce summary estimates (combined mean and 95% confidence interval (CI)), using the DerSimonian and Laird random effects model, (DerSimonian and Laird, 1986) with between-study variance pooled by histological subtype. This was used to account for inherent variability in the MR acquisition protocols and analysis techniques used across studies; an example is shown in the Inline Supplementary Materials (S1).

The above process was repeated after re-grouping the literature data into the four WHO grade groups (I-IV), and again after grouping all tumours into either low-grade (WHO I-II) and high-grade (III-IV) categories. Throughout this study, all group comparisons were performed using either *t-*tests (two groups) or their non-parametric equivalent for non-normally distributed data (Mann Whitney *U* test for un-paired samples, Wilcoxon rank sum tests for paired samples). Differences between multiple groups were identified using a one-way ANOVA test of subgroup summary measures (Borenstein et al., 2009). Post-hoc group comparisons were corrected for multiple comparisons using Tukey's honestly significant difference criterion (Tukey, 1949).

### 2.2 Cohort validation

#### 2.2.1 Patients

Paediatric patients with primary brain tumours seen at the neuro-oncology clinic at our hospital were retrospectively reviewed, after institutional ethical approval. Inclusion criteria were: patients who received ASL and DWI imaging between June 2015 (when ASL was introduced for paediatric brain tumours at our hospital) to September 2017, and patients with no prior resection, biopsy or treatment of the tumour.

#### 2.2.2 Magnetic resonance imaging

All patients were examined either at 3T (MAGNETOM Prisma, Siemens, Erlangen, Germany) or at 1.5 T (MAGNETOM Avanto, Siemens). On both scanners, ASL was performed using a prototype pseudo-continuous labelling sequence, with background suppression, and a 3D gradient-and-spin-echo readout. The labelling duration was 1800 ms, with a 1500 ms post-labelling delay, and ten repetitions were acquired. The DWI acquisition consisted of a diffusion-sensitised axial 2D spin-echo sequence with EPI readout, with a maximum b value of 1000 s/mm^2^. In addition, the MRI protocol included standard sequences for brain tumour investigations, including axial T2-weighted (T2w) imaging, axial T1w imaging pre- and post‑gadolinium contrast agent injection, and fluid attenuated inversion recovery (FLAIR) imaging. T2w, ASL and DWI sequences were acquired prior to the injection of gadolinium, and full details of these sequences are given in the Inline Supplementary Materials (S2).

#### 2.2.3 Post-processing

All post-processing, image analysis, and statistics were performed in Matlab (Mathworks Inc., Natick, MA) unless otherwise stated. Repetitions of the raw ASL images were checked for patient motion prior to averaging, and where necessary, individual mis-aligned volumes were corrected using an affine registration. All image registrations were performed using the *flirt* algorithm in FSL (FMRIB, Oxford, UK), using affine registrations (12 degrees of freedom) derived from a correlation ratio cost function.

CBF maps (units of ml blood / 100 g tissue / min) were calculated using the method described in Alsop et al. (2014) with λ = 0.9, α = 0.85, and T_1bl_ = 1.65 s. The ADC maps were produced directly by the scanner, and converted into standard units (mm^2^/s) prior to analysis using an in-house Matlab script. CBF and ADC maps were co-registered to the axial T2w scan. The ADC maps were registered directly to T2w scans; for the ASL data, the M_0_ calibration image was used to calculate the transformation matrix between the ASL and T2w scans, which was then applied to the CBF maps.

#### 2.2.4 Manual ROI placement

Whole-tumour ROIs were drawn by two readers (both consultant neuro-radiologists; KM (reader 1), 8 years of experience; Fd'A (reader 2), 4 years of experience). These were drawn around solid portions of the tumour (including enhancing and non-enhancing tissue) on the T2w axial images, using all available slices. Areas of cyst, haemorrhage, necrosis, and large blood vessels were avoided by cross-reference to the standard imaging sequences. The readers were blinded to the patient's histopathological diagnosis.

**Fig. 1:**
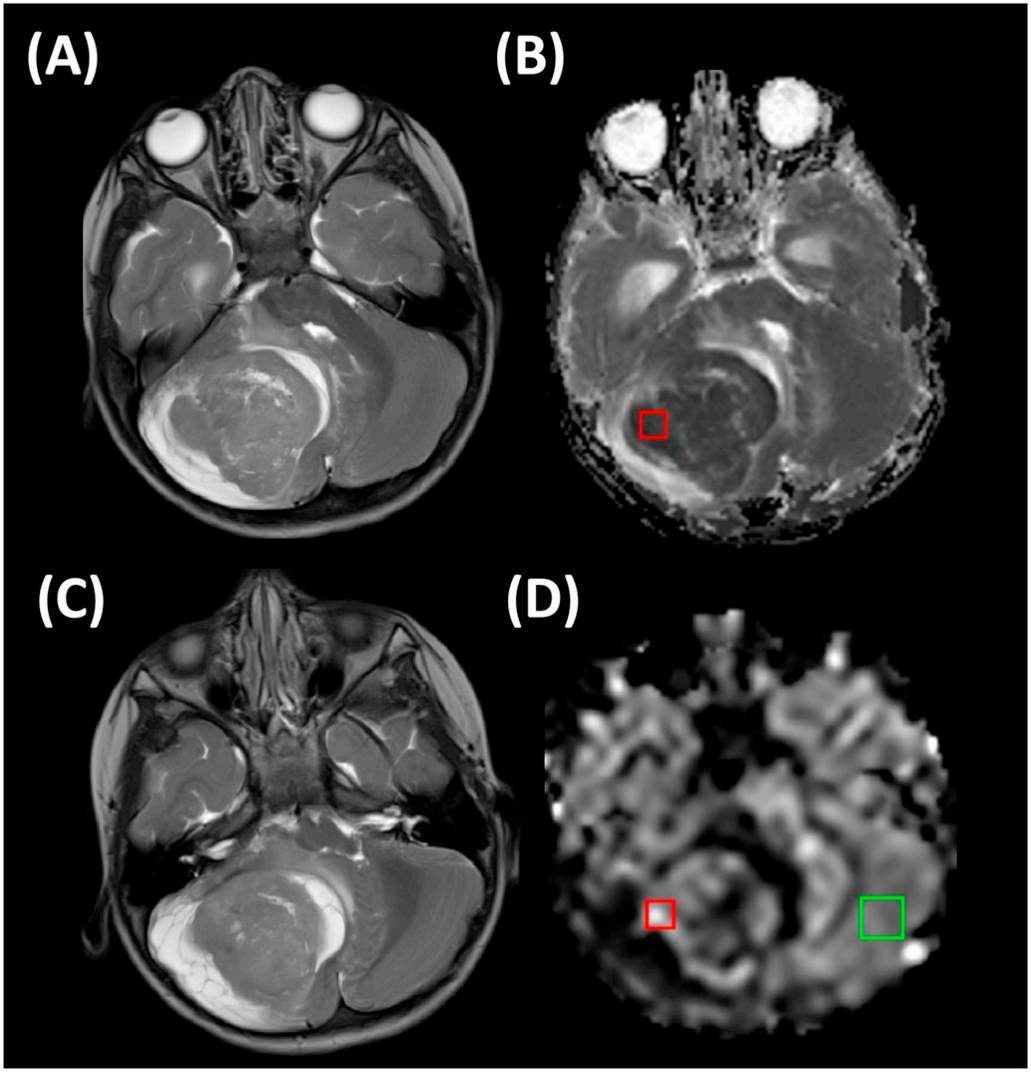
ADC and CBF ROI placement in an example patient (medulloblastoma, WHO IV). Axial T2w slices are shown in (A) and (C). The ADC map, and tumour ADC ROI placed by one of the readers (red square), are shown in (B). Similarly, the CBF map, with the tumour CBF ROI (red square), and normal appearing grey matter ROI (green square), placed by the same reader, are shown in (D).

Both readers then independently sampled each tumour using two ROIs, each square in size and 50mm^2^ in area, and fitting completely within the previously defined whole-tumour ROI. The size of this ROI was chosen to best match the ROI sizes used across the studies included in the meta-analysis, the average size of which was 48 ± 25 mm^2^ (in all studies where this was specified). One ROI was placed on the ADC map in the region of lowest-appearing ADC, the other on the CBF map in a region of highest-appearing CBF (both determined via visual inspection). An example is illustrated in Fig. 1. The mean value from the ADC ROI was used for ADC_ROI-mean_, the minimum value was used for ADC_ROI-min_, and the maximum value from the CBF ROI was used for CBF_ROI-max_. For CBF normalisation, a 150mm^2^ ROI was placed in normal-appearing contralateral grey matter (Fig. 1), similar to the technique described in Yeom et al. (2014).

#### 2.2.5 Automated ROI placement

In order to judge the performance of each reader in manually finding the lowest ADC and highest CBF tumour regions via visual inspection, every tumour was then automatically sampled using an in-house routine written in Matlab. For this, all possible locations for a 50mm^2^ ROI which fit wholly within the tumour (as defined by the whole-tumour ROIs drawn by the two readers) were identified. Of these, the optimal ADC ROI placement was chosen as the location with the lowest mean ADC within the ROI, and the optimal CBF ROI placement was chosen as the location with highest mean CBF. This represented the ‘gold standard’ for the placement of these ROIs, which cover regions that are thought to represent the most cellular or most vascular regions of the tumour respectively. For normalisation of the CBF values in this part of the study, the *FAST* algorithm (Zhang et al., 2001) in FSL was used to automatically segment the grey matter on the T2w images, and any overlap between the grey matter mask and tumour ROI was automatically excluded. The mean CBF value in grey matter in the axial slice at the mid-point of the tumour was used for normalisation (for automated CBF measurements only).

In addition, in order to determine the effect of ROI size on the quantification of tumour ADC and CBF values in our cohort, the automated ROI placement routine described above was reiterated, with ROI sizes of 25, 75, and 100 mm^2^. The lower limit of this range was chosen to ensure that any misalignment of the co-registered ADC and CBF maps, due to the unique distortions inherent in each sequence, did not outweigh the coverage of the ROI. Lastly, ADC_mean_, ADC_min_, and nCBF_max_ were measured over the entire tumour volume (based on the whole-tumour ROIs defined above) for comparison.

For analysis of the validation cohort, agreement between manual ADC and CBF measurements from the two readers was assessed using the intraclass correlation coefficient (ICC). In addition, the reproducibility coefficient (RPC) was defined as 1.96 x the SD of the difference in measured values across the cohort. Bias was defined as the mean difference between a reader's manual ADC or CBF measurements and the equivalent gold-standard values from automated evaluation. Optimum threshold values for separating low- and high-grade tumours in the validation cohort were calculated using receiver operator characteristic (ROC) analysis.

The combined predictive power of ADC and CBF measurements, for separating low/high grade tumours, was also examined. For this, ADC and CBF values from the same tumour were used as predictors in a logistic regression model, with high/low grade as the binary outcome. This was performed using Matlab's *fitlm* function, and the mathematical form of the model is given in the Inline Supplementary Materials (S4).

## 3 Results

### 3.1 Meta-analysis

A total of 14 studies met the inclusion criteria, constituting data acquired from 542 patients (9 studies reported ADC values (290 patients), 5 reported CBF values (252 patients)). Details are given in Table 1.

**Table 1:** Summary of the studies included in the meta-analysis. ADC_ROI-mean/min_, mean/min tumour apparent diffusion coefficient; nCBF_ROI-max_, maximum tumour cerebral blood flow, normalised to normal-appearing grey matter; pCASL, pseudo-continuous arterial spin labelling; PASL, pulsed arterial spin labelling; BL, bolus length; PLD, post-labelling delay; NS, not specified.

Study	Parameters measured	No. patients	Age range (years)	Magnetic Field Strength (T)	DWI b-values (s/mm^2^) or ASL modality (BL/PLD, ms)
Bull (2012)	ADC_ROI-mean_, ADC_ROI-min_	54	0.1–15.8	1.5	b = 0,500,1000
Calmon (2017)	ADC_ROI-mean_	18	3.3–14.7	1.5	b = 0,1000
Chang (2003)	ADC_ROI-mean_	6	1.1–15.0	1.5	b = 0,1000
Chen (2010)	ADC_ROI-mean_, ADC_ROI-min_	22	3.8 – NS	1.5	b = 0,1000
Choudhri (2015)	ADC_ROI-mean_	20	0.5–16.8	1.5, 3	b = 0,1000
Koral (2013)	ADC_ROI-mean_, ADC_ROI-min_	95	1.2–17.4	1.5, 3	b = 0,1000
Kralik (2014)	ADC_ROI-min_	19	0.08–1.0	1.5	b = 0,1000
Poretti (2013)	ADC_ROI-mean_	24	0.08–18.5	1.5	b = 0,1000
Rumboldt (2006)	ADC_ROI-mean_	32	0.1 - 23.0	1.5	b = 0,500,1000
Dangouloff-Ros (2016)	nCBF_ROI-max_	129	0.2–18.0	1.5	pCASL (NS/1025)
Dangouloff-Ros (2015)	nCBF_ROI-max_	13	0.6–16.0	1.5	pCASL (NS/1025)
Kikuchi (2017)	nCBF_ROI-max_	19	0.2–12.0	3	pCASL (1650/1525)
Morana (2018)	nCBF_ROI-max_	37	2.0–17.0	1.5	PASL (NS/1500-1800)
Yeom (2014)	nCBF_ROI-max_	54	0.2–18.0	3	pCASL (1500/1500)

#### 3.1.1 Tumour histological subtypes

**Fig. 2:**
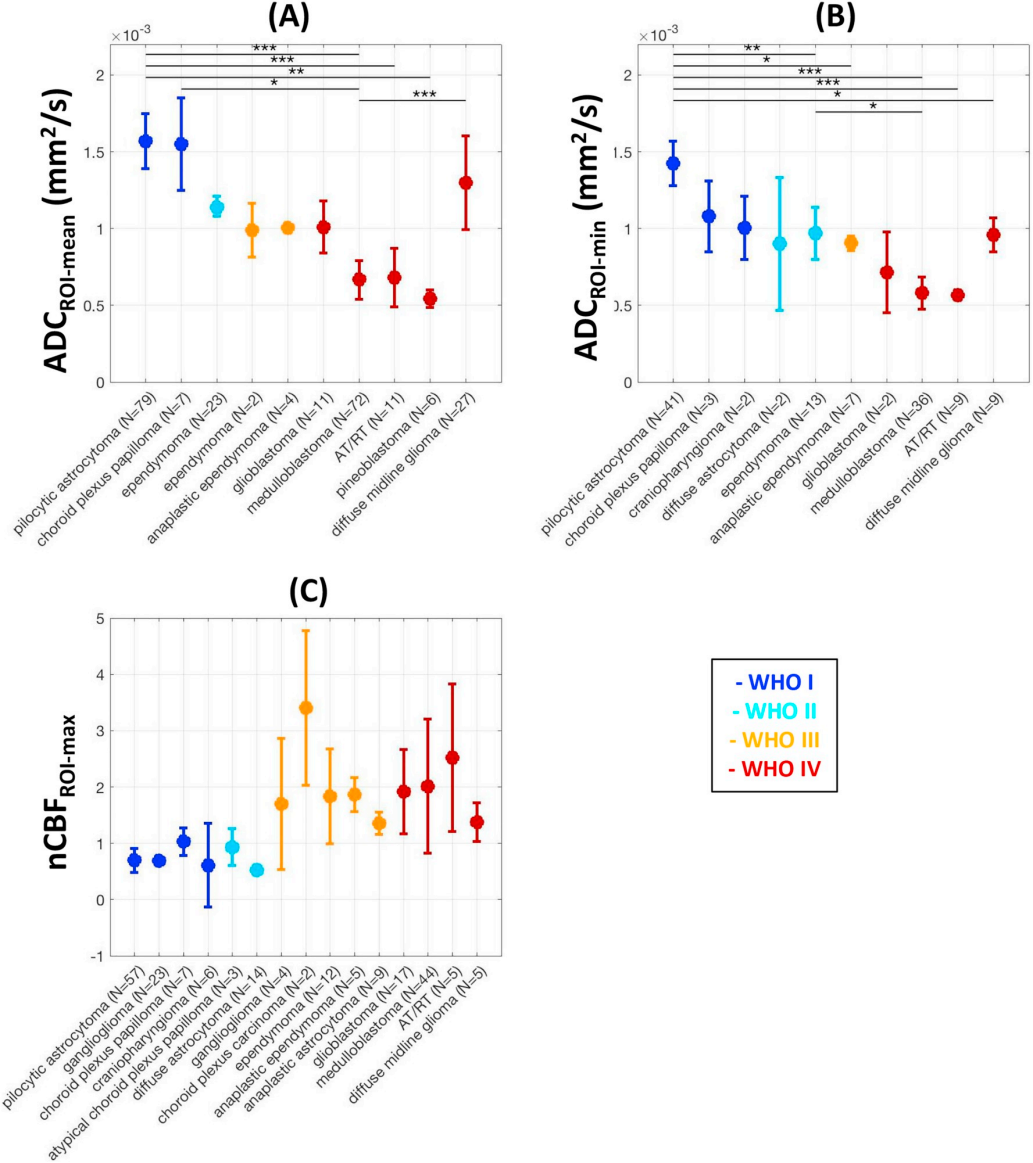
Summary estimates for (A) ADC_ROI-mean_, (B) ADC_ROI-min_ and (C) nCBF_ROI-max_ across histological brain tumour subtypes. Data points and error bars represent mean and 95% CI values for a given subtype respectively, after combination of data from the contributing studies using the random effects model. Colours represent WHO grade (see legend), and horizontal lines represent significant differences between subtypes after multiple comparison correction using Tukey's honestly significant difference criterion. N represents the total number of patients available for each subtype. * *p* < .05, ** *p* < .01, *** *p* < .001.

Summary estimates are shown in Fig. 2, which indicate the range of ADC and CBF values seen across different histological tumour subtypes, after combining the available data from the literature. For a given parameter, subtypes were included if data were available for 2 patients or more, after combination across the contributing studies. All significant differences between groups are indicated in the figure.

Although there was appreciable overlap between many of the histological subtypes, pilocytic astrocytoma had significantly higher ADC values compared to both medulloblastoma and atypical teratoid/rhabdoid tumour (AT/RT), using both ADC_ROI-mean_ and ADC_ROI-min_ values. In addition, diffuse midline glioma differed from other high-grade tumours, with ADC_ROI-mean/min_ and nCBF_ROI-max_ values more similar to those found in low-grade subtypes. No significant differences in nCBF_ROI-max_ values between subtypes survived multiple comparison correction.

#### 3.1.2 WHO tumour grades

**Fig. 3:**
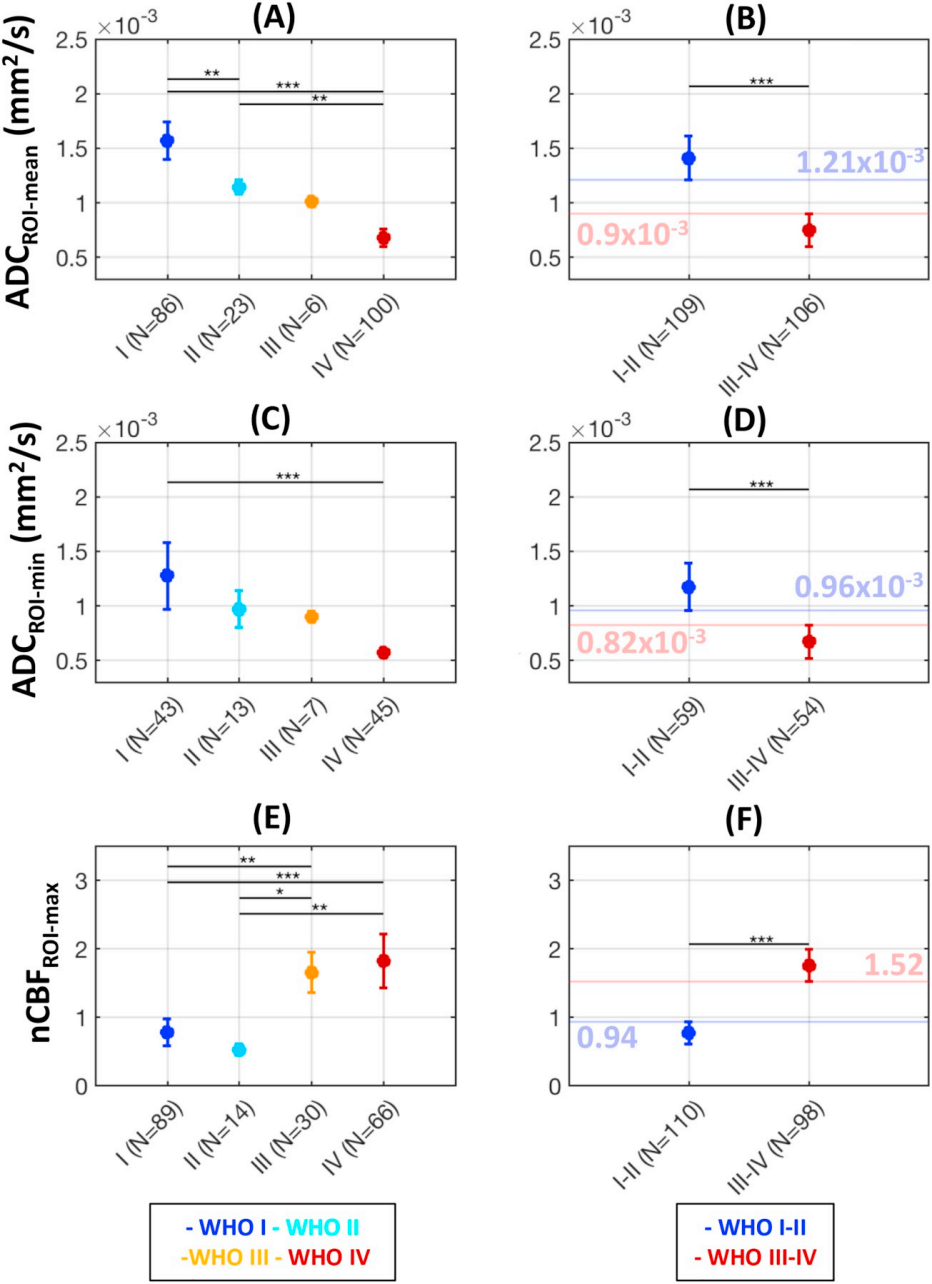
Summary estimates for ADC_ROI-mean_ (top row), ADC_ROI-min_ (middle row) and nCBF_ROI-max_ (bottom row). Tumours are categorised into individual WHO grade groups (left column), and into low-grade (I-II) and high-grade (III-IV) groups (right column). Blue and red lines (with values overlaid) in B, D, and F represent the bounds for potential threshold values which could best separate low- and high-grade lesions (see Section 3.2.3). Symbols are the same as those used in Fig. 2, with *N* representing the total number of patients available for each group. * p < .05, ** p < .01, *** p < .001.(For interpretation of the references to color in this figure legend, the reader is referred to the web version of this article.)

After re-grouping all tumours by WHO grade, summary estimates for each parameter are shown in Fig. 3. Also shown are the summary estimates after re-grouping tumours into low-grade (WHO I-II) and high-grade (WHO III-IV) categories.

Overall, as the WHO grade of a tumour increased, ADC values decreased and CBF increased. A one-way ANOVA with post-hoc multiple comparison correction revealed improved stratification across WHO grades I to IV using ADC_ROI-mean_ values (*p* = 8.8 × 10^−19^) compared to ADC_ROI-min_ (*p* = 5.7 × 10^−5^). nCBF_ROI-max_ values also demonstrated good separation between individual grades (*p* = 1.2 × 10^−7^).

After re-grouping tumours into low-grade and high-grade categories, both ADC_ROI-mean_ and ADC_ROI-min_ were significantly higher in low-grade vs. high-grade tumours (*p* = 5.8 × 10^−7^ and 3.7 × 10^−4^ respectively), and nCBF_ROI-max_ was significantly lower in low-grade tumours (*p* = 8.2 × 10^−11^). The boundaries for the ‘target area’ for potential threshold values for optimal separation of low- and high-grade tumours, defined as the region between the 95% CIs of the two groups, are illustrated in Fig. 3 (see also Section 3.2.3).

### 3.2 Cohort validation

The inclusion criteria were fulfilled by 32 patients (17 females). The median age at pre-treatment MRI was 4.8 years (range 0.4 to 14.5 years). Histologic examination revealed the following subtypes (WHO grade in brackets): 10 pilocytic astrocytomas (I), 1 pilomyxoid astrocytoma (I), 2 gangliogliomas (I), 2 anaplastic ependymomas (III), 7 diffuse midline gliomas (1 x III, 5 x IV, 1 not biopsied), 6 medulloblastomas (IV), 2 glioblastomas (IV), 1 atypical teratoid/rhabdoid tumour (IV), and 1 mixed germ cell tumour (IV).

#### 3.2.1 Manual ROI placement – inter-reader agreement and comparison with automated assessment

The agreement in manual measurements of tumour ADC and CBF values between the two readers is shown in Table 2. Also shown is bias (mean difference) between each reader's manual measurements and the gold-standard values obtained via automated evaluation.

**Table 2:** Inter-reader agreement for manual measurement of the parameters of interest, and comparison of each reader's measurements to automated parameter evaluation. AE, automated evaluation; ICC, intraclass correlation coefficient; RPC, reproducibility coefficient.

	Reader 1 vs. Reader 2		Reader 1 vs. AE		Reader 2 vs. AE	
Parameter	ICC	RPC	ICC	Bias (R1-AE)	ICC	Bias (R2-AE)
ADC_ROI-mean_	0.96	0.33 × 10^−3^ mm^2^/s	0.84	7.80%	0.83	10.50%⁎
ADC_ROI-min_	0.95	0.31 × 10^−3^ mm^2^/s	0.67	12.31%⁎	0.62	12.08%
CBF_ROI-max_	0.97	24.88 ml/100 g/min	0.62	−15.72%	0.60	−21.44%⁎⁎
CBF_GM_	0.95	19.06 ml/100 g/min	0.87	13.52%⁎⁎	0.85	13.74%⁎⁎
nCBF_ROI-max_	0.94	1.33	0.64	−22.33%⁎	0.64	−21.07%⁎

⁎ *p* < .05

⁎⁎ *p* < .01

Between the two readers, all manually measured parameters showed excellent correlation across the range of tumours in our cohort, with ICCs of 0.94 or higher. The limits of agreement were slightly better for ADC values compared to CBF; after normalising to the mean measured value for each parameter, the RPC values shown in Table 2 represent an inter-reader variability of 31% and 36% for ADC_ROI-mean_ and ADC_ROI-min_ values, and 44% and 42% for CBF_max_ and mean CBF in the grey matter (CBF_GM_), respectively. As nCBF_ROI-max_ represents the quotient of two independently measured values (CBF_max_ and CBF_GM_), the RPC value was accordingly high: the value of 1.33 represents 82% of the mean nCBF_ROI-max_ values across the cohort.

Overall, the manually measured parameter values showed moderate correlation with the equivalent ‘gold standard’ automated values; ADC_ROI-mean_ showed the best correlation (mean ICC of 0.84), and CBF_max_ the poorest (mean ICC of 0.61). In general, manual measurements of ADC_ROI-mean_, ADC_ROI-min_, and CBF_GM_ were over-estimated compared to the gold standard, and CBF_ROI-max_ and nCBF_ROI-max_ were under-estimated (see bias values in Table 2).

#### 3.2.2 Subtype comparisons

Due to the limited size of the validation cohort, only the following subtypes could be considered for between-group comparisons: pilocytic astrocytoma (*N* = 10), diffuse midline glioma (*N* = 7) and medulloblastoma (*N* = 6). All other subtypes had fewer than 3 patients. In the meta-analysis, it was suggested that pilocytic astrocytomas should be separable from medulloblastomas using ADC_ROI-mean_ or ADC_ROI-min_. It was also suggested that diffuse midline glioma should be separable from medulloblastoma using ADC_ROI-mean_, and will appear more similar to pilocytic astrocytoma.

Plots of the between-group differences for the above histological subtypes are shown in the Inline Supplementary Material (S3). Using automated values from our cohort, the pilocytic astrocytomas did indeed demonstrate significantly higher ADC_ROI-mean_ and ADC_ROI-min_ values than the medulloblastomas (*p* = 9.5 × 10^−5^ and *p* = 2.2 × 10^−4^ respectively, ANOVA with post-hoc multiple comparison correction). In addition, the diffuse midline gliomas demonstrated significantly higher values of ADC_ROI-mean_ and ADC_ROI-min_ compared to the medulloblastomas (*p* = .0014 and *p* = .0030 respectively). In our cohort, the medulloblastomas also demonstrated significantly higher nCBF_ROI-max_ values than both the pilocytic astrocytomas (*p* = .012) and diffuse midline gliomas (*p* = .011). Lastly, as suggested by the meta-analysis, no significant differences were found between the pilocytic astrocytomas and diffuse midline gliomas, in any of the parameters.

#### 3.2.3 Low- and high-grade tumour separation

**Fig. 4:**
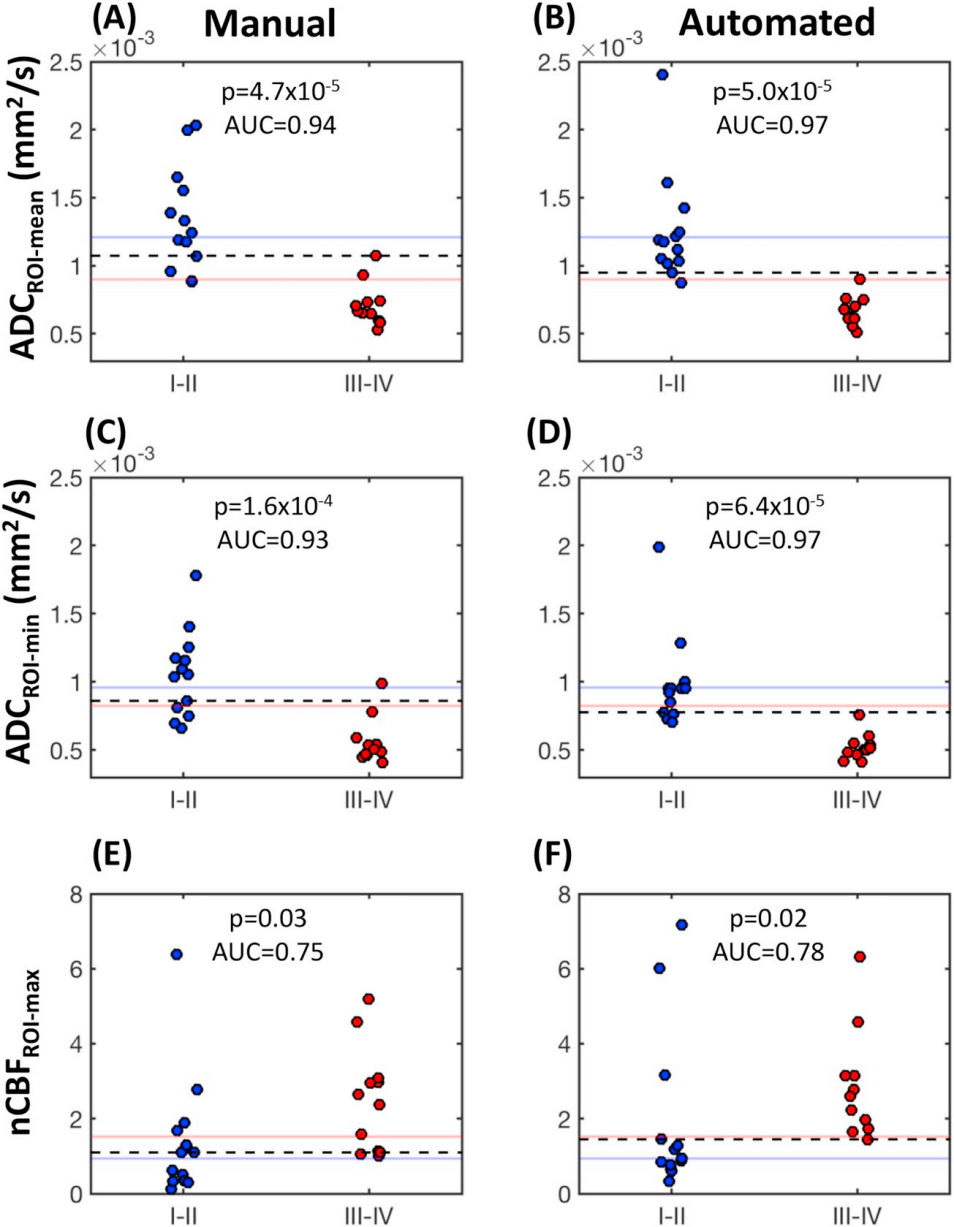
Separation of low-grade (WHO I-II) and high-grade (WHO III-IV) tumours, using ADC_ROI-mean_ (top row), ADC_ROI-min_ (middle row) and nCBF_ROI-max_ (bottom row). Data points are derived from manual ROI measurements (left column) and automated ROI measurements (right column). The threshold value for optimum separation of high/low grade tumours in the validation cohort, derived from ROC analysis, is shown as a dashed line for both manual and automated values. For comparison, the red and blue horizonal lines represent the anticipated bounds of the ‘target region’ for these threshold values, derived from the meta-analysis (Fig. 3). Between-group *p*-values obtained from two-sided Wilcoxon rank sum tests, and area under the curve (AUC) values from ROC analysis, are indicated in each panel. (For interpretation of the references to color in this figure legend, the reader is referred to the web version of this article.)

After re-grouping the tumours in our cohort into low-grade (WHO I-II) or high-grade (WHO III-IV) categories, group differences in ADC_ROI-mean_, ADC_ROI-min_, and nCBF_ROI-max_ are shown in Fig. 4. Diffuse midline gliomas were excluded (N = 7), due to the aforementioned atypical features of these high-grade tumours. The positions of the optimal threshold values for separation of the two groups, derived from ROC analysis, are also shown in Fig. 4, relative to their suggested ‘target region’, as suggested by the findings from the meta-analysis.

The AUC values shown in Fig. 4 indicated that automated measurements provided improved separation of low/high grade tumours, compared to manually derived values. Using the automated values, the optimum ADC_ROI-mean_ threshold for low/high-grade separation was 0.95 × 10^−3^ mm^2^/s, proving a 96% accuracy in low/high-grade classification. For ADC_ROI-min_, this was 0.78 × 10^−3^ mm^2^/s (83% accuracy); and for nCBF_ROI-max_, 1.45 (83% accuracy). All but one of the threshold values shown in Fig. 4 fell within the ‘target regions’ suggested by the meta-analysis. The only exception was the automated ADC_ROI-min_ threshold – our cohort suggested a value just below the lower bound suggested by the meta-analysis (0.82 × 10^−3^ mm^2^/s, Fig. 4D). However, applying the latter to our cohort gave equally good separation between the two groups, and as such a threshold of 0.82 × 10^−3^ mm^2^/s for ADC_ROI-min_ is likely to be optimal.

**Fig. 5:**
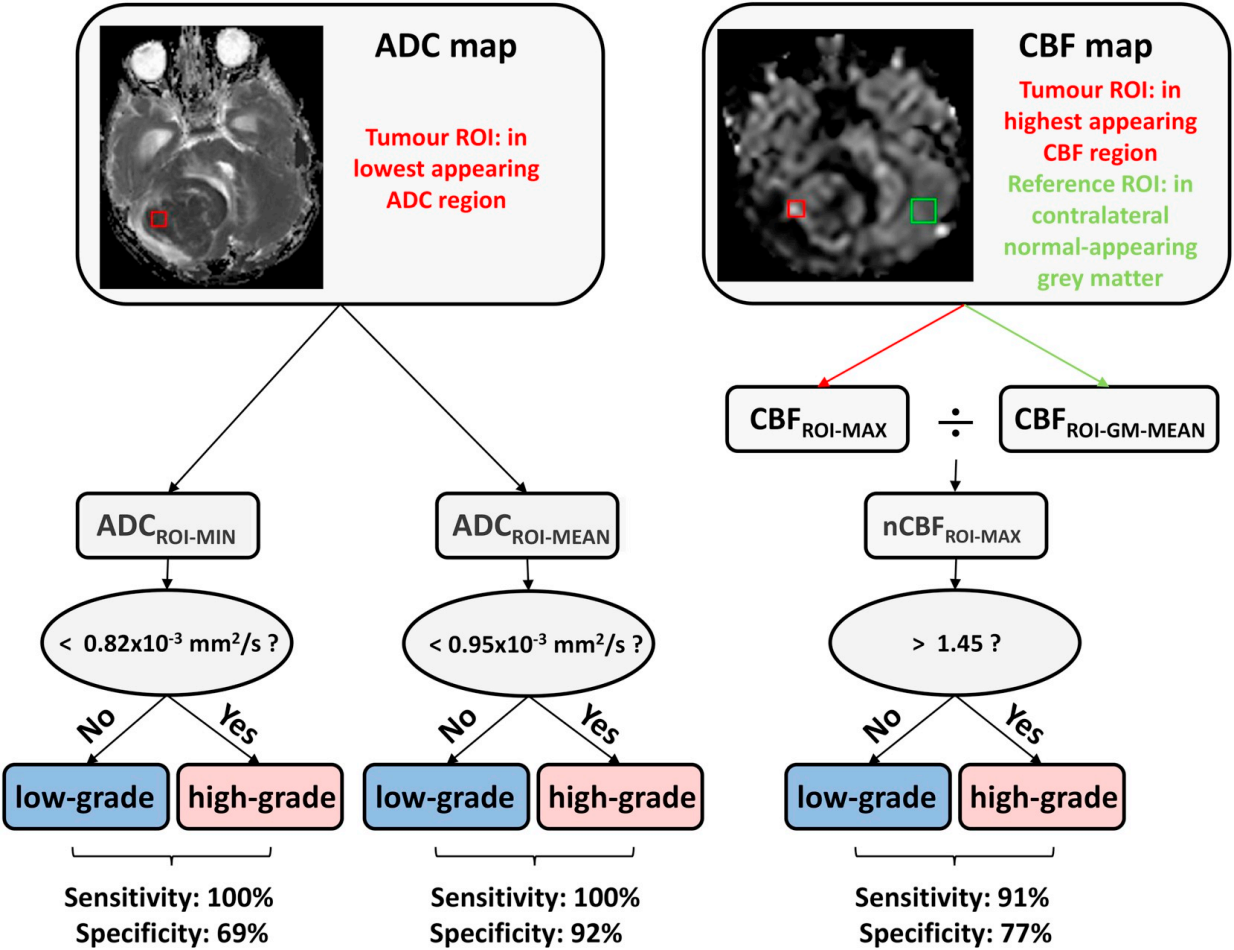
Flowchart illustrating the use of ADC_ROI-min_, ADC_ROI-mean_ or nCBF_ROI-max_ values for differentiating low- and high-grade paediatric brain tumours. ADC_ROI-min/mean_ represent the minimum/mean values from the ROI placed on the ADC map, and nCBF_ROI-max_ is the maximum value from the ROI placed on the CBF map, divided by the mean CBF value from the ROI placed in contralateral grey matter. The above thresholds are suitable for most histological subtypes, with the exception of diffuse midline glioma. Additionally, a subset of low-grade pilocytic astrocytoma may present with nCBF_ROI-max_ values above 1.45.

Overall, ADC values provided superior separation of low/high grade tumours, compared to CBF. This was largely due to a sub-set of low-grade glial tumours which demonstrated markedly high CBF (two pilocytic astrocytomas, with CBF_max_ of 85 and 149 ml/100 g/min; and one pilomyxoid astrocytoma with CBF_max_ of 149 ml/100 g/min; Fig. 4F; see Discussion). A flowchart summarising the use of these thresholds for identifying low- and high-grade tumours, and their estimated sensitivity and sensitivity based on their application in our validation cohort, is illustrated in Fig. 5.

**Fig. 6:**
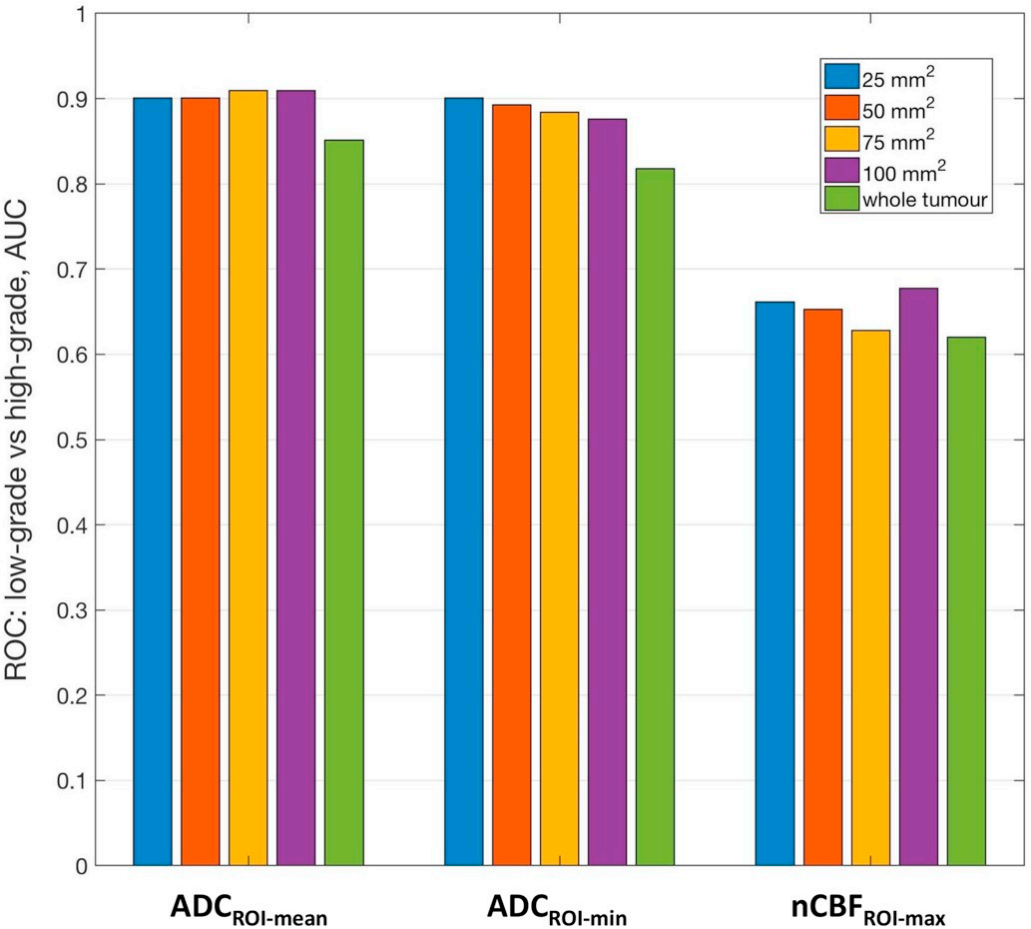
Plots of the AUC values derived from ROC analysis of low-grade vs. high-grade tumour stratification, based on ADC and CBF measurements obtained from the automated placement of ROIs with a range of sizes (25–100 mm^2^). Also shown are the AUC values after using ADC and CBF measurements obtained from ‘whole tumour’ ROIs.

Lastly, the effect of changing the size of the ROI used for the automated ADC and CBF measurements is shown in Fig. 6. In terms of separation of low/high grade tumours, varying the ROI size between 25 and 100 mm^2^ had only a minimal effect on the AUC values obtained from the ROC analysis (see Fig. 6); over this range, the coefficient of variation in AUC values was only 0.5% for ADC_ROI-mean_, 1.2% for ADC_ROI-min_, and 3.2% for nCBF_ROI-max_. However, when the ‘whole tumour’ ROI was used, particularly for ADC_ROI-mean/min_, the AUC values were markedly lower than those obtained with the smaller ROIs (25 to 100 mm^2^, Fig. 6).

#### 3.2.4 Combined use of ADC and CBF as predictors

**Fig. 7:**
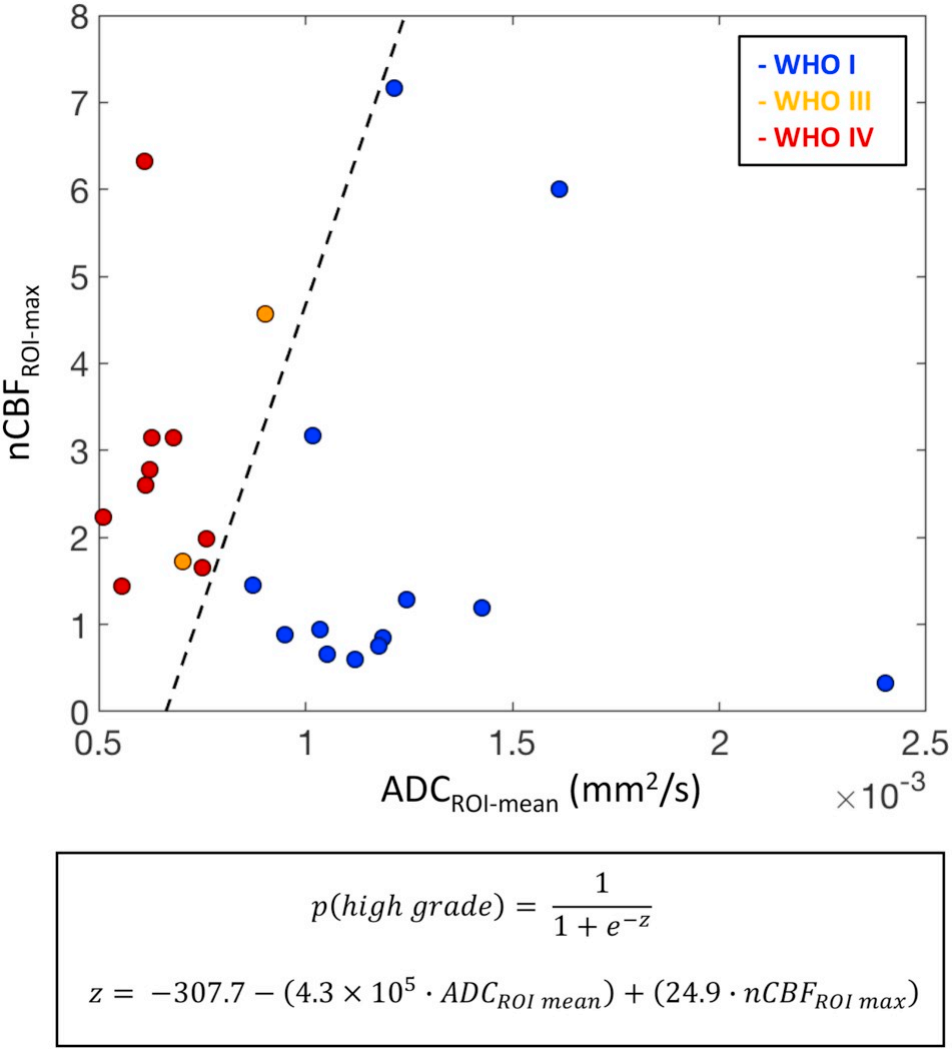
Separation of low-grade (WHO I-II) and high-grade (WHO III-IV) tumours in the validation cohort, using automated ADC_ROI-mean_ and nCBF_ROI-max_ values as combined predictors in a logistic regression model. The dashed line represents the point of separation between the two groups (i.e. points at which the probability of tumour being high-grade and low-grade are both equal to 0.5). The mathematical expression of the model, including values for the fitted logistic regression coefficients, are given in the lower inset. Diffuse midline gliomas are excluded.

Using a logistic regression model, with automated values of either ADC_ROI-mean_ or ADC_ROI-min_ in combination with nCBF_ROI-max_ as independent variables, we were able to achieve correct low/high-grade classification in 100% of our tumours (again, after exclusion of diffuse midline glioma). An example is shown in Fig. 7 (the equivalent plot for the ADC_ROI-min_ + nCBF_ROI-max_ model is shown in the Inline Supplementary Materials, S4). As such, the predictive power of using ADC and CBF values combined was superior to either parameter used in isolation, albeit only marginally so in the case of using ADC_ROI-mean_ alone in our cohort.

Lastly, the logistic regression model described above, which was derived using automated ADC and CBF values, was applied to the manual measurements from each reader in turn. Using combined ADC_ROI-mean_ and nCBF_ROI-max_ values, the model correctly classified low/high grade status for 92% of the tumours for reader 1, and 96% for reader 2. Using combined ADC_ROI-min_ and nCBF_ROI-max_ values, the model correctly classified low/high grade status for 88% of the tumours for reader 1, and 92% for reader 2.

## 4 Discussion

Paediatric brain tumours are rare, and encompass a wide spectrum of biological subtypes. As such, it is challenging to define their quantitative imaging characteristics, such as typical ADC and CBF values, using data from a single institution. The aim of this study was to collate a body of evidence from previously published data in order to overcome this limitation. The included studies acquired data over a range of MRI scanners, pulse sequences, and field strengths (1.5–3 T). Accordingly, these data were combined using a random effects model, to account for between-study variations in methodology. As such, the resulting typical parameter ranges and recommendations presented here should be applicable over the range of MRI scanners and acquisition protocols typically encountered in the clinic. By demonstrating the concordance of the results from the meta-analysis with data acquired in a new cohort of patients, we aimed to demonstrate the applicability of these guidelines in a single-centre setting. Lastly, the image analysis techniques used in this study were limited to those which could be performed using typical clinical workstations with limited image processing capabilities, to assess the accuracy and reproducibility of a relatively simple quantification method which could be readily applied in the clinic.

### 4.1 Stratification of histological subtypes

After combing data across studies, our results suggest that considerable overlap in ADC and CBF values remains, and as such, many histological subtypes will not be separable using these parameters alone. However, both the meta-analysis and our validation cohort confirm that pilocytic astrocytomas have markedly higher ADC values compared to high-grade embryonal tumours such as medulloblastoma.

The results from the meta-analysis and the validation cohort also suggest that diffuse midline glioma is an ‘outlier’ among high-grade tumours, being characterised by ADC and CBF values which are more similar to low-grade glial tumours. This is perhaps surprising given that diffuse midline gliomas carry the worst prognosis of all childhood brain tumours. However, this poor prognosis is largely due to the location of these tumours in the brain stem, and their highly infiltrative nature. These tumours intermix with surrounding white matter tracts, and despite the conspicuity of these lesions on T2w imaging, it has been proposed that only small areas of signal abnormality represent anaplastic tumour tissue (Lobel et al., 2011). This makes separating malignant tumour tissue from surrounding parenchyma problematic, and as such, ADC and CBF values are likely to be ‘corrupted’ by the admixture of tumour and non-tumour tissue. It has been suggested that regions which demonstrate T2 hypo-intensity in conjunction with postcontrast signal enhancement and diffusion restriction may represent focal anaplasia in these tumours, (Lobel et al., 2011) which may alleviate this issue in future studies.

### 4.2 Stratification of low- and high-grade tumours

As illustrated in Fig. 4, the threshold values for differentiating low- and high-grade tumours presented here were in good agreement with the results from the meta-analysis. Furthermore, our suggested ADC_ROI-mean_ threshold of 0.95 × 10^−3^ mm^2^/s is in good agreement with the value of 0.9 × 10^−3^ mm^2^/s suggested by Orman et al. (2015), and the mean difference in nCBF_ROI-max_ between the low- and high-grade groups of 0.99 in our study falls within the 95% CI range reported recently in Delgado et al. (2018)

Our analysis suggests that ADC values provide superior separation of low-grade and high-grade tumours, compared to CBF. As mentioned above, this was due to a subset of low-grade glial tumours with markedly high CBF. Previous studies have shown that, despite their low-grade nature, histologically, pilocytic astrocytomas demonstrate a high vascular density (Birlik et al., 2006). However, the delivery of blood to these tumours appears to remain generally low: both our validation cohort, and data from the meta-analysis, confirm that the majority of pilocytic astrocytoma tumours are hypo-perfused. There are, however, a minority in which markedly high blood flow is observed, which may get over-looked when summary values are reported. Further studies to investigate the clinical progression of these highly perfused pilocytic astrocytomas, as compared to their hypo-perfused counterparts, would be of interest. Nonetheless, once ADC and CBF values are combined, all pilocytic astrocytomas were correctly classified as either low- or high-grade in our cohort, demonstrating that diagnostic utility is maximised by combining ADC and CBF values in the same tumour.

In addition, our results suggest that sampling a sub-section of the tumour, via either manual or automated placement of a small ROI, provided superior stratification of low/high-grade tumours, compared to values drawn from the entire solid tumour region. Our results were approximately consistent using ROIs in the range of 25-100 mm^2^, all of which provided improved stratification compared to the whole-solid-tumour ROIs.

### 4.3 ADC_ROI-mean_ or ADC_ROI-min_?

The results from both the meta-analysis and the validation cohort suggest that overall, the performance of the ADC_ROI-mean_ and ADC_ROI-min_ parameters was approximately equivalent; however, ADC_ROI-mean_ performed marginally better in a small number of cases (improved ICC with automated values, improved performance in the meta-analysis in terms of WHO grade stratification, and marginally higher AUC values for low/high-grade tumour separation in our cohort). However, it should be noted that the mean / min ADC values used in this study do not generally refer to ‘whole-tumour’ mean or minimum values. Rather, the ADC_ROI-mean_ and ADC_ROI-min_ parameters refer to the distribution of values within an ROI, placed in an area of solid tumour judged to have comparatively low ADC via visual or automated assessment. The mean value derived from this area is likely to provide a more stable estimate of local cellularity, compared to the minimum value, which is ultimately decided by a single voxel.

### 4.4 Reliability of the manual placement of ROIs for ADC and CBF quantification

A further aim of this study was to evaluate the reliability of ADC and CBF measurements when ‘done by hand’, by experienced radiologists. This arguably represents the most realistic manner by which these measurements are obtained clinically, where image analysis infrastructure is often limited, and is also representative of the techniques used in the studies included in the meta-analysis.

Our results suggest that ADC and CBF values obtained by two independent readers show excellent correlation across a range of common paediatric brain tumour subtypes and grades. However, the absolute values varied between readers. As CBF_max_ must be normalised to normal-appearing grey matter, the measurement of which itself is subject to variability between readers, the final values of nCBF_ROI-max_ can be highly variable when performed manually.

It is clearly challenging, and subjective, for a reader to pick out the lowest ADC or highest CBF region of a tumour ‘by eye’, and the automated evaluation of these ROI placements suggest that, typically, manually measured ADC values are overestimated, and nCBF_ROI-max_ underestimated. Measures derived from the automated method afforded more accurate separation of tumour subtypes when compared to the manually derived measures, and as such our findings support a move towards automated tumour sampling where possible. However, when using the logistic regression model (with ADC and CBF values combined), manually measured values provided a low/high-grade predictive accuracy of 92% for reader 1 and 96% for reader 2. As such, the limited precision and accuracy of manually measured values do not appear to greatly diminish their diagnostic utility, and their use is justified if automated tumour sampling techniques are not available.

### 4.5 Limitations

Our study had a number of limitations. Firstly, the size of our validation cohort was fairly small, and as such we were not sufficiently powered to validate a number of the histological subgroup comparisons presented in the meta-analysis. This reflects a general limitation of performing a single-centre study on a rare disease with a broad spectrum of biological features. Furthermore, we did not account for molecular tumour classifications in this study, which is likely to form a key aspect of diagnosis in future paediatric brain tumours studies. However, a sufficient quantity of data regarding ADC and CBF values in these paediatric molecular subgroups has not yet been reported in the literature to allow for a meaningful meta-analysis.

In addition, the large size of ROI used for deriving CBF values in normal-appearing grey matter, in relation to the typical thickness of cortical grey matter, may result in CBF values derived from these regions being subject to partial volume effects with nearby white matter or CSF. Furthermore, the algorithm used for automated segmentation of grey matter for CBF normalisation purposes may have been impaired by the presence of a tumour. Although visual inspection of the segmentations indicated this was not a major problem in this study, the automated grey matter CBF values may be slightly inaccurate due to this issue. Lastly, we did not consider more complex metrics regarding the distribution of ADC or CBF values within a tumour, such as percentile values, skewness, etc. Although previous studies have shown utility in some of these parameters, our aim in this study was to examine imaging metrics which could be readily measured in a clinical environment, with limited image processing capabilities.

## 5 Conclusion

The available data regarding ADC and CBF in paediatric brain tumours indicate these parameters are useful for the stratification of certain histological subtypes, and, where possible, the significant differences between subtypes identified in the literature were confirmed in our validation cohort. Identification of low- and high-grade tumours is possible using ADC or CBF, and the threshold values presented here are in agreement with previously published studies and new data from an independent validation cohort. Automated tumour sampling methods provide improved accuracy and precision in the measurement of ADC and CBF, which in turn improves the diagnostic power of these parameters. This will be improved further when CBF and ADC values are combined to predict the malignancy of a tumour.

## Appendix A Supplementary data

Supplementary material Image 1
