# Supplementary material for: Arterial spin labelling and diffusion-weighted imaging in paediatric brain tumours
Source: Neuroimage Clin. 2019 Jan 29;22:101696. doi: 10.1016/j.nicl.2019.101696 (PMC6365981; doi:10.1016/j.nicl.2019.101696)
Supplement: Supplementary file 1 — Supplementary material [file mmc1.docx]

**Inline Supplementary Material**

**S1 Calculation of summary effect sizes**

For a given histological subtype (or WHO grade), mean parameter values from each contributing study were combined, with weights assigned to each study using a random effects model, in order to produce an overall summary effect size. An example for pilocytic astrocytoma (WHO grade I) is shown in Figure S1.


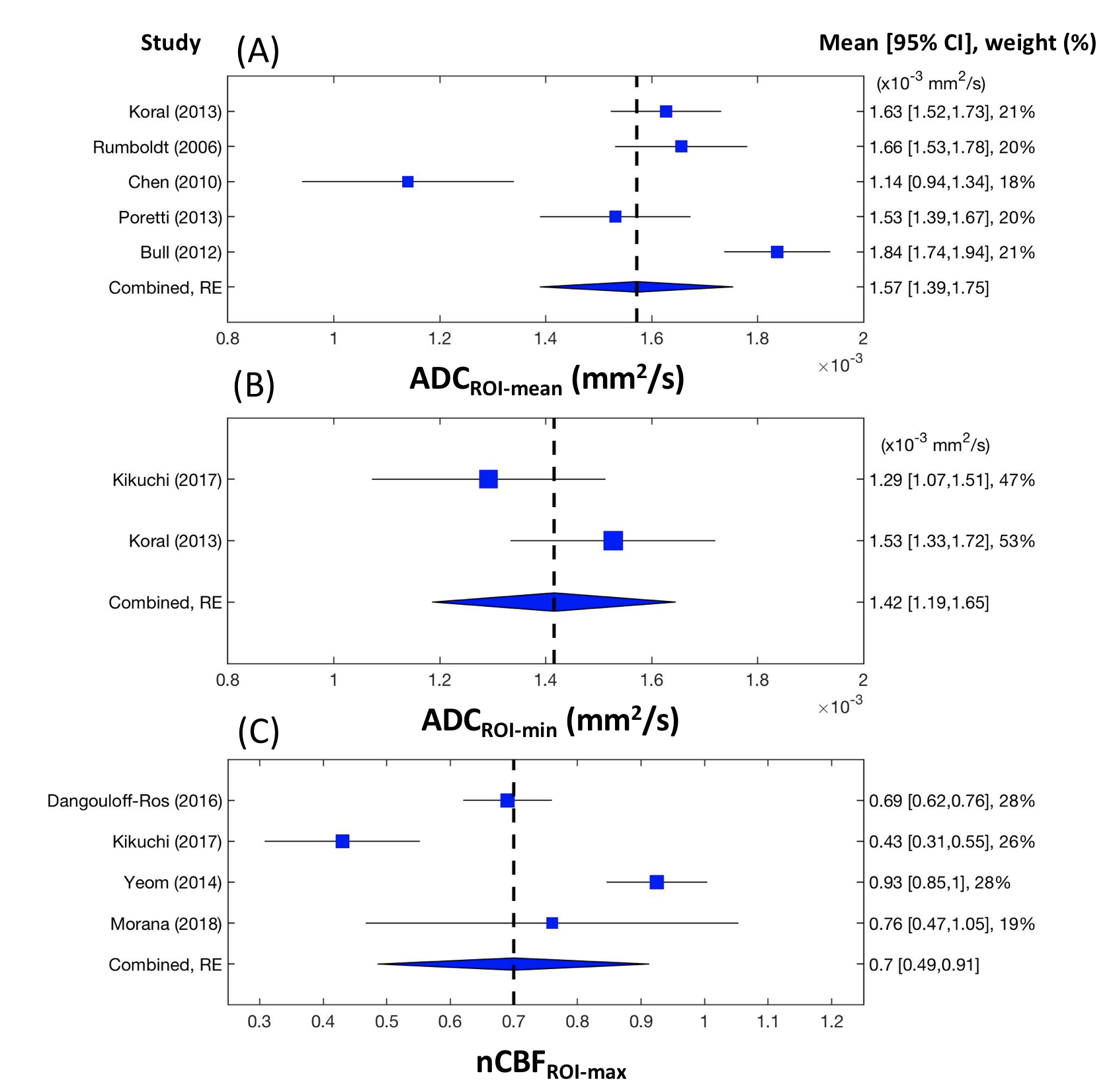


**Figure S1** Example of the combination of (A) ADC_ROI-mean_, (B) ADC_ROI-min_, and (C) nCBF_ROI-max_ values in pilocytic astrocytoma across all contributing studies. Mean study values are shown as squares, with horizontal bars representing 95% CIs. The summary effect size is shown as a filled diamond, with the mean value represented as a vertical line, and the 95% CI represented by the diamond width.

**S2. MR sequence parameters for ASL, DWI, and T2-weighted acquisitions**

Sequence parameters for the ASL, DWI and T2-weighted acquisitions are summarised in Table S1, and additional, sequence-specific information is given below.

|  | **ASL** | | **DWI** | | **T2** | |
| --- | --- | --- | --- | --- | --- | --- |
|  | **1.5 T** | **3 T** | **1.5 T** | **3 T** | **1.5 T** | **3 T** |
| Field of view (mm) | 220 | 220 | 230 | 230 | 220 | 230 |
| Matrix | 64 x 62 | 64 x 62 | 128 x 128 | 192 x 192 | 384 x 306 | 448 x 448 |
| In-plane resolution (mm) | 3.4 x 3.5 | 1.7 x 1.7^a^ | 1.8 x 1.8 | 1.2 x 1.2 | 0.7 x 0.6 | 0.5 x 0.5 |
| No. slices / partitions | 24 | 24 | 19 | 25 | 34 | 30 |
| Slice thickness (mm) | 5.0 | 4.0 | 5.0 | 4.0 | 4.0 | 4.0 |
| TR (ms) | 4160 | 4620 | 2700 | 3600 | 6690 | 5930 |
| TE (ms) | 17.8 | 21.8 | 96 | 59 | 101 | 85 |

**Table S1** Summary of sequence parameters. ^a^ interpolation factor 2.

For the ASL acquisition, a prototype pseudo-continuous labelling sequence with background suppression, and a 3D gradient-and-spin-echo (GRASE) readout was used. Additional sequence parameters were: labelling duration was 1800ms, with a 1500ms post-labelling delay, turbo factor=12, EPI factor=31, segments=2 (3 T, with parallel imaging, GRAPPA=2), 4 (1.5 T, no parallel imaging), repetitions = 10; acquisition time = 2 min 39 s (1.5 T), 3 min 19 s (3 T). A proton-density weighted (M_0_) image was also acquired (TR=4000ms), with identical readout to the ASL acquisition but with the labelling RF pulses removed, for CBF quantification. In order to minimise artefacts from through-plane blurring inherent in the 3D GRASE sequence, the echo train length used for the pCASL sequences was kept below 300 ms.

For the DWI acquisition, a diffusion-sensitised axial 2D spin-echo sequence with EPI readout was used. This was acquired using a single-shot at 1.5 T, and using a readout-segmented multi-shot EPI acquisition (Siemens resolve sequence) at 3 T. Additional sequence parameters were: b-value = 0, 500, 1000 s/mm^2^ (1.5 T), 0, 1000 s/mm^2^ (3 T), applied in 3 orthogonal directions.

The T2-weighted acquisition consisted of an axial turbo spin-echo sequence. Additional sequence parameters were: turbo factor = 13 (1.5 T), 32 (3 T); flip angle = 150° (1.5 T), 120° (3 T); GRAPPA = 2 (3 T); acquisition time = 4 min 22 s (1.5 T), 2 min 59 s (3 T).

**S3 Between-group comparisons of histological subtypes in the validation cohort**

Group differences in ADC_ROI-mean_, ADC_ROI-min_, and nCBF_ROI-max_ values (derived from automated ROI placement), in the pilocytic astrocytomas (N=10), diffuse midline gliomas (N=7) and medulloblastomas (N=6), are illustrated in Figure S2.


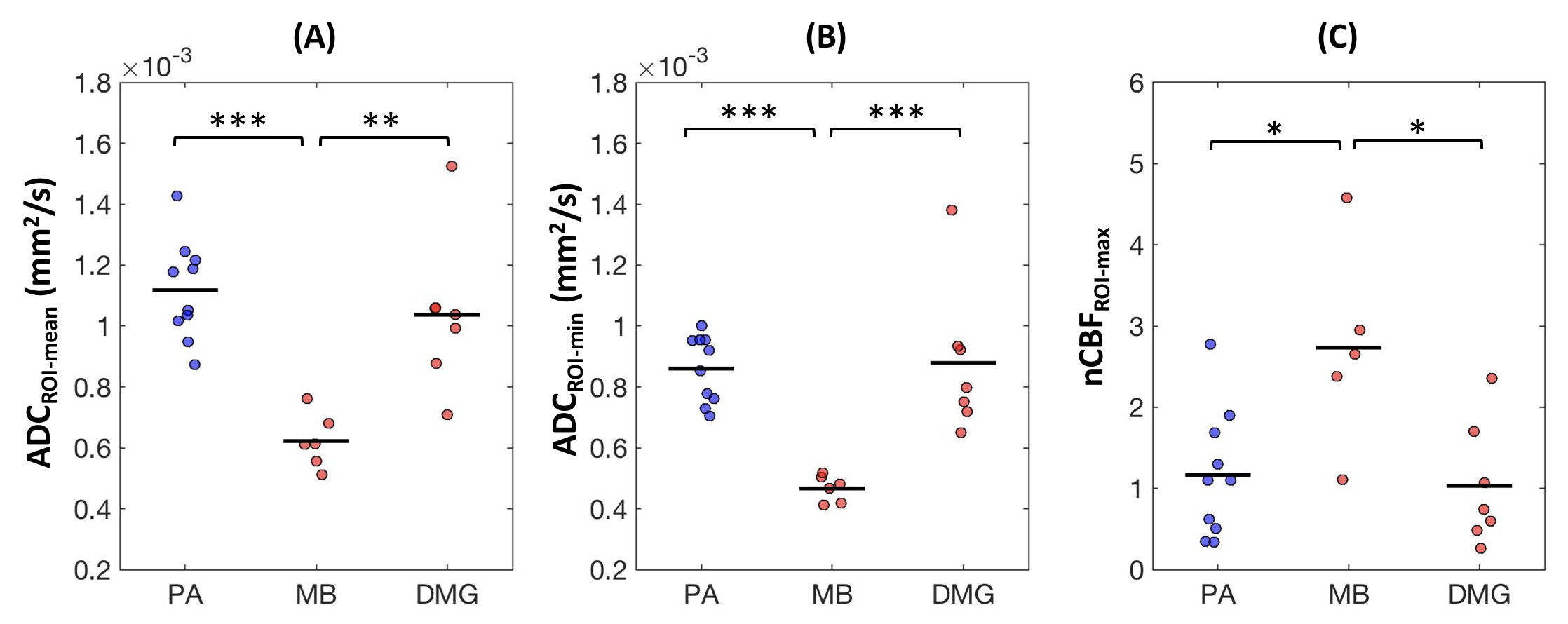


**Figure S2** Group comparison of (A) ADC_ROI-mean_, (B) ADC_ROI-min_, and (C) nCBF_ROI-max_ values in pilocytic astrocytoma (PA), medulloblastoma (MB) and diffuse midline glioma (DMG). Values are derived from automated ROI placements in the validation cohort. * p < 0.05, ** p < 0.01, *** p < 0.001.

**S4 Logistic regression model for the combined use of ADC and CBF as predictors of tumour grade**

The logistic regression model used to classify low- and high-grade tumours, based on a combination of their ADC and CBF values, took the following form:

| $p\left( high grade \right)= \frac{1}{1+e^{-z}}$ | (1) |
| --- | --- |

where

| $z= \beta_{0}+\left( \beta_{1}\cdot ADC \right)+\left( \beta_{2}\cdot\mathrm{nCBF}_{\max} \right)$. | (2) |
| --- | --- |

Here, p(high grade) represents the probability of a tumour being high-grade (0 to 1; the probability of the same tumour being low-grade is defined as 1-p(high grade)); ADC is either ADC_ROI-mean_ or ADC_ROI-min_; β_0_ is the fitted intercept term, and β_1,2_ are fitted coefficient estimates for ADC and nCBF_ROI-max_ values respectively. If p(high grade) > 0.5, the tumour was classified as high-grade, otherwise it was classified as low-grade.

Automated ADC and CBF values were used to test this model. The fitted values of β_0_, β_1_, β_2_ were 307.7, -4.3x10^5^, and 24.9 using ADC_ROI-mean_ as a the ‘ADC’ predictor, and 351.5, -5.4x10^5^, and 13.6 using ADC_ROI-min_. Both versions of this model provided correct low/high grade classification in 100% of the tumours in our validation cohort, as illustrated in Figure 7 in the main paper (using ADC_ROI-mean_ + nCBF_ROI-max_), and Figure S3 below (using ADC_ROI-min_ + nCBF_ROI-max_).


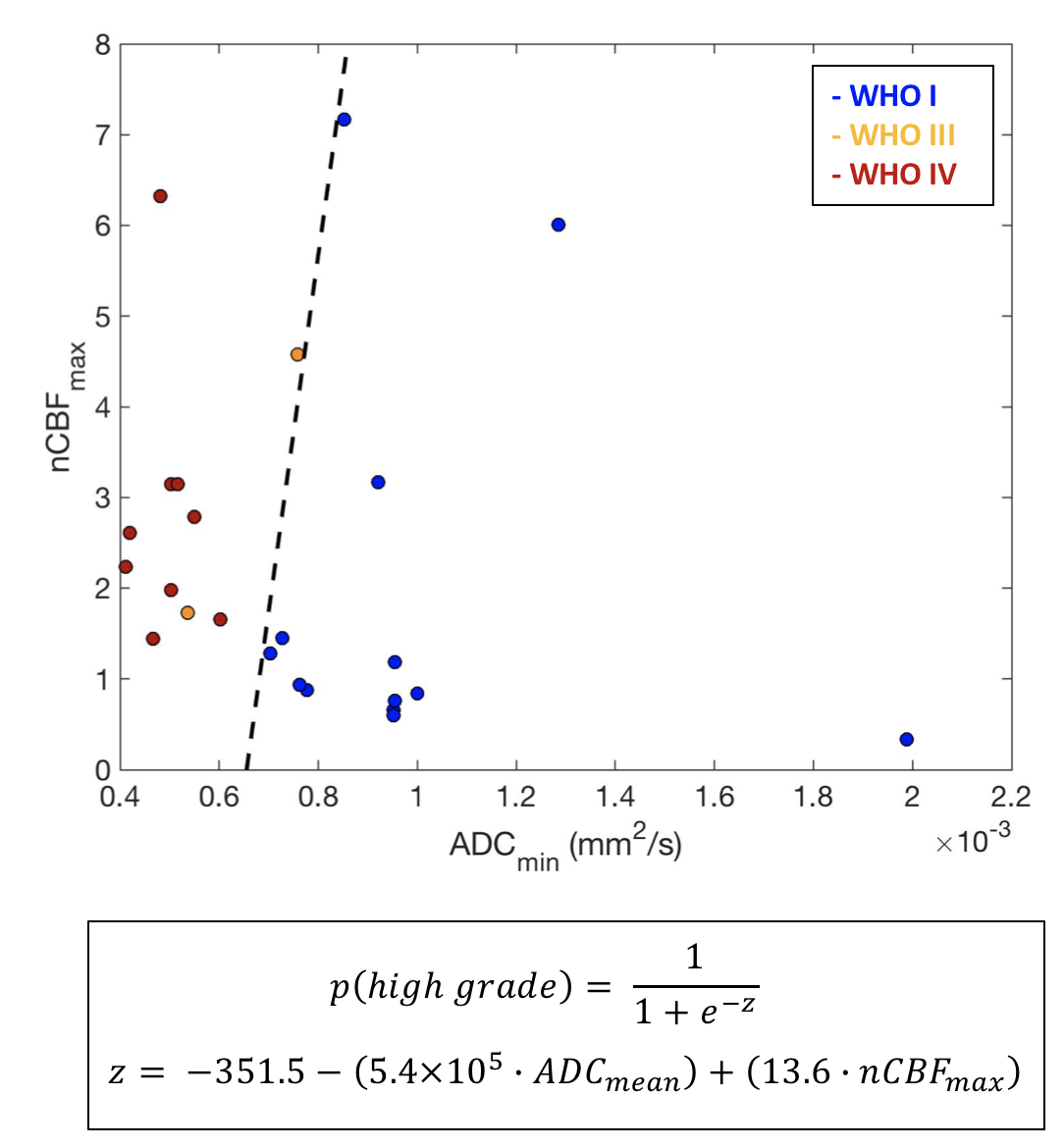


**Figure S3** Separation of low-grade (WHO I-II) and high-grade (WHO III-IV) tumours in the validation cohort, using automated ADC_ROI-min_ and nCBF_ROI-max_ values as combined predictors in a logistic regression model. The dashed line represents the point of separation between the two groups (i.e. points at which the probability of tumour being high-grade and low-grade are both equal to 0.5). The mathematical expression of the model, including values for the fitted logistic regression coefficients, are given in the lower inset. Diffuse midline gliomas are excluded.
